# Editorial: Reviews in: ophthalmology 2023

**DOI:** 10.3389/fmed.2024.1464594

**Published:** 2024-07-25

**Authors:** Georgios D. Panos, Rashmi Deshmukh

**Affiliations:** ^1^Department of Ophthalmology, AHEPA University Hospital, School of Medicine, Faculty of Health Sciences, Aristotle University of Thessaloniki, Thessaloniki, Greece; ^2^Division of Ophthalmology and Visual Sciences, School of Medicine, Faculty of Medicine and Health Sciences, University of Nottingham, Nottingham, United Kingdom; ^3^Cornea and Refractive Surgery Service, LV Prasad Eye Institute, Kallam Anji Reddy Campus, Hyderabad, India

**Keywords:** ophthalmology, reviews, retina, cornea, cataract, strabismus and pediatric ophthalmology

## Introduction

Welcome to the Research Topic, “*Reviews in: ophthalmology 2023*.” As we step into another year of exploration and discovery in the field of ophthalmology, this Research Topic is dedicated to encapsulating the pivotal advancements, enduring challenges, and future trajectories that define our current understanding of ocular health and disease. The year 2023 marks another milestone in the relentless pursuit of knowledge, driven by innovative research and technological breakthroughs that continue to transform the landscape of eye care.

In this Research Topic, we have meticulously curated a series of reviews that encompass a broad spectrum of ophthalmological research, from the latest in retinal therapy and glaucoma management to cutting-edge surgical techniques and pediatric ophthalmology developments. These reviews provide an insightful snapshot of contemporary knowledge, emphasizing the progress achieved and the obstacles that remain.

Each review in this Research Topic offers a unique and comprehensive perspective, underscoring the depth and diversity of current ophthalmic research. From assessing the efficacy of novel therapeutic approaches and surgical advancements to dissecting the genetic intricacies of ocular diseases, these articles represent the forefront of ophthalmic science and clinical practice.

As we present “*Reviews in: ophthalmology 2023*,” our aim is to enhance the understanding of the current ophthalmological landscape, inspire continued research, and ultimately contribute to the advancement of patient care. We invite you to explore these thought-provoking reviews, expand your knowledge, and join us in the ongoing journey to understand the complexities of the human eye.

## Summaries of articles

Ożóg et al. provide an in-depth review of the epiretinal membrane (ERM), a pathological tissue formed at the vitreoretinal interface, which can cause significant vision disturbances. Their article explores the etiology, epidemiology, pathophysiology, and treatment of ERM, highlighting the various cell types and cytokines involved in its formation.

Guo et al. conducted a meta-analysis to evaluate subclinical changes in corneal dendritic cell density (CDCD) and corneal subbasal nerve density (CSND) in asymptomatic contact lens wearers. Their findings indicate an increase in CDCD among contact lens wearers, while no significant difference was observed in CSND, underscoring the utility of *in vivo* confocal microscopy (IVCM) for such assessments.

Qu et al. delve into the complexities of neovascular glaucoma, a condition often resulting from central retinal vein occlusion that can lead to blindness. Their review discusses the pathogenesis of the disease and evaluates the efficacy of pan-retinal photocoagulation and intravitreal anti-VEGF injections, recommending a combined approach for better long-term outcomes.

Wang et al. assess the efficacy and safety of intrastromal lenticule implantation for hyperopia correction through a comprehensive meta-analysis. They report significant improvements in uncorrected distance visual acuity and spherical equivalent refractive outcomes, while calling for further research on corneal biomechanics and long-term safety.

Yeh et al. review the etiologies and management of childhood blindness in West Africa, a critical global health issue affecting millions of children. Their study highlights treatable causes such as cataracts and vitamin A deficiency, emphasizing the need for ongoing research to standardize reporting and implement effective public health measures.

Lin et al. (a) examine the rotational stability of toric intraocular lenses (IOLs), which are designed to correct corneal astigmatism. They discuss various factors influencing postoperative rotation and advocate for a personalized approach to minimize rotation and enhance visual outcomes.

Gong et al. review the complications associated with Implantable Collamer Lens (ICL) surgery, particularly those affecting intraocular pressure (IOP). They provide detailed insights into common and rare complications, such as residual viscoelastic and Toxic Anterior Segment Syndrome (TASS), emphasizing prevention and early diagnosis.

Zhang et al. explore the impact of exercise and physical activity on various ocular diseases. Their review highlights the benefits of exercise on conditions like dry eye disease, cataracts, and glaucoma, and discusses mechanisms such as improved blood circulation and reduced oxidative stress, advocating for further research into exercise-based therapeutic strategies.

Gan et al. provide a systematic review of complications associated with XEN gel stent implantation, a minimally invasive procedure for glaucoma. They identify common and rare complications and stress the importance of vigilant postoperative monitoring and early intervention.

Lentz et al. review the effects of hyperbaric conditions, such as those experienced during SCUBA diving, on intraocular pressure (IOP). They find that increased atmospheric pressure generally reduces IOP, though the underlying mechanisms remain unclear. The authors highlight the potential of hyperbaric chambers for glaucoma treatment, while noting the need for further research.

Lin et al. (b) investigate anterior capsular contraction syndrome (ACCS), a complication that can occur after cataract surgery, affecting visual outcomes. Their review covers the pathogenesis, clinical course, and management of ACCS, calling for more research to develop optimal prevention and intervention strategies.

The reviews compiled in this Research Topic not only highlight the latest advancements and therapeutic strategies but also address the ongoing challenges and gaps in our understanding of ocular diseases. We hope that these reviews will inspire further research and innovation, ultimately leading to improved care for patients with ocular diseases. As we look toward the future, it is clear that the work of today's researchers and clinicians will pave the way for tomorrow's breakthroughs in eye care. We encourage you to dive into these insightful reviews, learn something new, and get involved in the exciting developments in ophthalmological research. By staying informed and engaged, we can all help improve vision health and the quality of life for those affected by eye diseases. Thank you for your dedication to ophthalmology, and we eagerly anticipate the progress and discoveries to come.

## Author contributions

GP: Writing – original draft, Supervision, Methodology. RD: Writing – review & editing.

